# Hydrogen Sulfide, Oxidative Stress and Periodontal Diseases: A Concise Review

**DOI:** 10.3390/antiox5010003

**Published:** 2016-01-14

**Authors:** Maria Greabu, Alexandra Totan, Daniela Miricescu, Radu Radulescu, Justina Virlan, Bogdan Calenic

**Affiliations:** Dental Medicine Faculty, Biochemistry Department, University of Medicine and Pharmacy CAROL DAVILA, 8 Blvd EroilorSanitari, sect.5, 050474 Bucharest, Romania; mariagreabu@yahoo.com (M.G.); miricescudaniela@yahoo.com (D.M.); radu_radulescu24@yahoo.com (R.R.); roxxanajustina@yahoo.com (J.V.); bcalenic@yahoo.co.uk (B.C.)

**Keywords:** hydrogen sulfide, saliva, periodontitis

## Abstract

In the past years, biomedical research has recognized hydrogen sulfide (H_2_S) not only as an environmental pollutant but also, along with nitric oxide and carbon monoxide, as an important biological gastransmitter with paramount roles in health and disease. Current research focuses on several aspects of H_2_S biology such as the biochemical pathways that generate the compound and its functions in human pathology or drug synthesis that block or stimulate its biosynthesis. The present work addresses the knowledge we have to date on H_2_S production and its biological roles in the general human environment with a special focus on the oral cavity and its involvement in the initiation and development of periodontal diseases.

## 1. Introduction

Hydrogen sulfide (H_2_S), traditionally recognized as a toxic gas with a rotten-egg smell [1], is also a bacterial waste product eliminated in the subgingival pocket [2,3]. Periodontal disease has been described as an immune-inflammatory condition characterized by connective tissue breakdown, loss of attachment and alveolar bone resorption [4]. In periodontitis pathogenesis, inflammatory and immune reactions play the main roles [5], but more and more authors consider also the link between oxidative stress and periodontal problems. Due to H_2_S’s abilities in reducing oxidative stress [6,7,8] or regulating inflammation [8,9], researchers have started studying H_2_S’s roles in the initiation and progression of periodontal diseases. However, results are controversial.

Interestingly, H_2_S can be regarded as a double-faced molecule: on one side, at lower concentration have antioxidant and cytoprotective activities, but at higher concentrations is cytotoxic and stimulates oxidative stress (OS).

This paper reviews the most significant studies concerning H_2_S production, its biological roles and implications in periodontitis development.

## 2. H_2_S—Production

**Figure 1:**
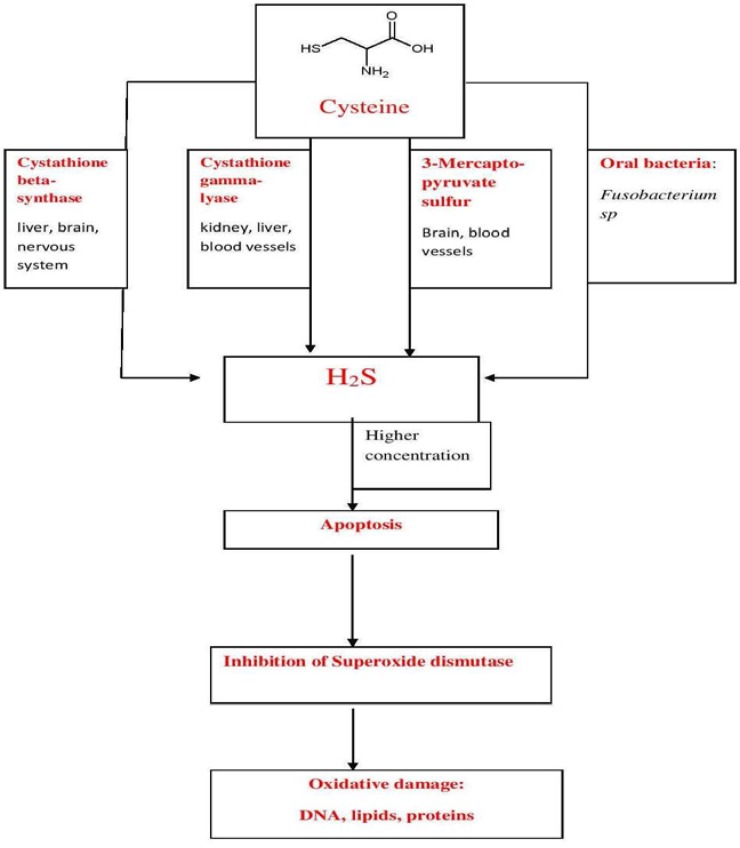
Hydrogen sulfide production—Cysteine biosynthesis pathway is the main pathway responsible for H_2_S production in mammalian organisms. It usually needs the help of three oral enzymes.

In mammalian organisms, including the human body, endogenous H_2_S synthesis is generally connected to three enzymes: 3-mercaptopyruvate sulfurtransferase (3MST), cystathionine β-synthase (CBS) and cystathionine γ-lyase (CSE), all three taking part in the cysteine synthesis pathway [10,11,12] (Figure 1).

It is important to note that the three enzymes are responsible for H_2_S homeostasis and regulate H_2_S levels found in the bloodstream. Each of the enumerated enzymes is found at specific sites in the organism. Thus, 3MST has a mitochondrial location and is usually present in the brain and blood vessels. The enzyme participates in a series of chemical reactions that starts with cysteine metabolism transformed to 3-mercaptopyruvate by cysteine aminotransferase. Further 3-mercaptopyruvate is reacted to pyruvate and, finally, to H_2_S by 3MST. CBS is encountered mostly in hepatic, cerebral and nervous tissues. CBS produces H_2_S as a result of a reaction involving cystathionine generation from cysteine and serine. Similarly, CSE, which resides in blood vessels and hepatic cells, produces H_2_S starting from cysteine and generates pyruvate and ammonia. H_2_S catabolism involves several oxidative steps that convert the gas to persulfide, thiosulfate and sulphate, in an organ specific process. Further, H_2_S oxidation was shown to occur in virtually all cell types and tissues of the human body including colon, kidney, liver, and brain or lung cells [13].

In the oral cavity, human periodontal ligament stem cells (PDLSCs) express H_2_S-synthesizing enzymes CSE and (CBS) [14]. CBS may be the main source of endogenous H2S in PDLSCs [14]. H_2_S is also caused by the metabolic products of oral sulfate-reducing bacteria [15] that degrade substrates such as cysteine, arginine or tryptophan. Interestingly, gut bacteria produced H_2_S is considered a pathogenic factor in bowel inflammatory diseases characterised by inflammatory mucosal lesions like periodontal diseases lesions [16].

## 3. H_2_S Biological Roles

H_2_S is a gaseous mediator with multiple roles depending on the tissue or organ. Thus, H_2_S is involved in blood vessels dilatation, inflammation, cardiac reaction to ischemic injuries [17], nervous system regulation [18], insulin secretion, and resistance [1,19]. In the human body, increased concentrations of H_2_S are associated with respiratory affections such as chronic bronchitis, emphysema, pneumonia or diseases related to the cardiovascular system (e.g., hypertension) [17]. However, there is also a rapidly expanding body of evidence for essential roles of H_2_S in the protection against tissue injury, in reducing inflammation, and tissue repair [20]. H_2_S might be both beneficial and harmful in cerebral ischemic injury depending on its concentration [21]. A recent study found that the amino-oxyacetic acid (AOAA), an inhibitor of H_2_S synthesis, administered at a low dose has protective effects; but it worsens the ischemic injury at higher concentrations [21].

Among the most studied molecular mechanisms of H_2_S cellular effects is the regulation of intracellular redox homeostasis and post-translational modification of proteins through glutathione (GSH) generation and S-sulfhydration [1]. Moreover, H_2_S exerts anti-oxidative, anti-inflammatory and cytoprotective effects [22]. Sodium hydrosulfide (NaHS) (a H_2_S donor) had interesting effects in the kidneys of uranium-intoxicated rats: it managed to lower malondialdehyde(MDA) accumulation, and to restore GSH levels and anti-oxidative enzymes’ activities like superoxide dismutase (SOD), glutathione peroxidase (GPx), catalase (CAT) and glutathione S-transferase (GST) [22]. Furthermore, a mitochondrially targeted hydrogen sulfide donor exerts protective effects in renal epithelial cells subjected to OS, as it might be related to the reduction of cellular OS [23]. Also, H_2_S can protect neurons and cardiac muscle from OS and ischemia-reperfusion injury [24], as well as accelerating wound healing in diabetic animals [8].

Likewise, other volatile sulfur compounds related to H_2_S (*i.e.*, dimethyl sulfide) were shown to be significantly elevated in patients with cerebrovascular pathology (for example, subarachnoid or intracerebral hemorrhages), as well as increased cholesterol levels, asthma or hepatic affections like cirrhosis [25] (Table 1).

**Table 1:** Hydrogen sulfide—systemic effects.

Biological Event	H_2_S—Effect
Angiogenesis	Increases blood flow Decreases the risk of tissue injury
Mitochondrial respiration	Decreases the function Cytoprotection
Vasodilatation	Regulates blood pressure
Leukocyte adhesion	Anti-inflammatory effect
Apoptosis	Decreases apoptosis—cytoprotective effect
Antioxidant	Up-regulation of antioxidant molecules

Also, not surprisingly, several studies have focused on the H_2_S toxicity in the or alenvironment (Table 2 and Table 3).

**Table 2:** Biological effects of high physiological concentrations of H_2_S on different oral cell types (50ng/mL H_2_S).

Tissue	Cells	Origin	Biological Event
**Oral Epithelia**	Normal keratinocytes	Ca9-22 cell line	Apoptosis—mitochondrial pathway activated; DNA damage
	Keratinocyte stem cells	Human skin cell line	Apoptosis—mitochondrial pathway activated; DNA damage; p53 and Bax activity increased
	Keratinocyte stem cells	Human oral mucosa	Apoptosis—mitochondrial pathway activated; DNA damage; Activation of genes from p53 pathway connected with DNA repair, cell cycle arrest
	Keratinocyte cells	Animal oral mucosa	Increases the permeability of the epithelium
**Oral Dermis**	Fibroblasts	Human oral mucosa	Apoptosis—mitochondrial pathway activated; DNA damage
	Collagen Fibers	Extracellular matrix	Increases collagen degradation/decreases collagen synthesis
**Dental Pulp**	Dental pulp stem cells	Human dental pulp	Apoptosis—mitochondrial pathway activated; DNA damage
**Bone**	Osteoblasts	Mouse calvaria	Apoptosis—mitochondrial and death ligand pathway activated; DNA damage; Bone resorption

**Table 3:** Biological effects of low physiological concentrations of H_2_S on different oral cell types (1ng/mL H_2_S).

Tissue/Cells		Origin	Biological Event
Dental Pulp	Dental pulp cells	Human pulp	Differentiation to hepatic like cells
	Dental pulp cells	Human pulp	Differentiation of pancreatic like cells
Bone	Osteoclasts	Mouse	Osteoclast activation followed by bone resorption

## 4. Oxidative Stress and Periodontal Diseases

Reactive oxygen species (ROS) are products of normal oxygen metabolism and have beneficial biological effects, in low levels and under normal conditions. Instead, higher concentrations present harmful effects to the body. External environment (heat, UV light, X and gamma radiations, therapeutic drugs), behavioural activities (smoking, chronic exercise) and inflammatory cells (such as activated macrophages and neutrophils release various ROS (H_2_O_2_, NO, O_2_^−^, HO and HOCl) [26,27,28,29]. Even though ROS have extremely short half-lives, they can cause substantial damage to tissues and cellular components. Recently, systemic OS was also associated with the suppression of bacterial-specific IgG levels [30]. At the cellular level, ROS progression starts with membrane lipid peroxidation followed by cytosolic proteins modification and ending with DNA oxidation [31]. Lipid peroxidation is initiated by the hydroxyl radical, while its major final products are MDA and 4-hydroxyl-2-nonenal (HNE). Therefore, MDA is one of the most used biomarkers to evaluate oxidative damage in both local and systemic disorders [31].

Several amino acids (such as tyrosine) can also react with ROS, generating a wide range of products, from modified and less active enzymes to denatured, non-functioning proteins. Furthermore, mitochondrial DNA are also affected by the ROS attack. HO can react with all components of DNA molecules, damaging both purine and pyrimidine [26,27,28,32,33,34].

There are many enzymatic antioxidant defence mechanisms in order to protect against ROS effects *in vivo* [35]: SOD, GPx, CAT [35,36,37]. Saliva has its own fighting mechanisms: uric acid, ascorbate, reduced glutathione and alpha tocopherol [38,39,40,41,42]. Urate, the most important salivary antioxidant, acts as a scavenger for hydroxyl radical, singlet oxygen, or peroxynitrite, especially in presence of ascorbic acid or thiols [43]. Other sources of antioxidants in the oral cavity are albumin, catalase-positive commensal and fresh blood extravasated from injured capillaries [42]. More than that, SOD has been localized in the human periodontal ligament, and it is a valuable defence enzyme within gingival fibroblasts [35,44].

Tissue destruction in periodontal diseases is considered to be the result of an altered inflammatory/immune response to microbial plaque and involves massive release of neutrophils, ROS and enzymes [45,46,47,48]. Gingival epithelial cells form the first line of defence in the gingival crevice. So, they have the key role as the protection mechanism of host oral structures from bacterial invasion. Thus, gingival epithelial cells produce an adaptive immune responses [49] and release the chemotaxis factor for neutrophils [50,51], antimicrobial peptides [52] and pro-inflammatory cytokines, such as interleukin-8 (IL-8). Unfortunately, on the other hand, over-expression of these pro-inflammatory cytokines causes collateral tissue damage. ROS produced by activated neutrophils in response to periodontopathogenic bacteria cause serious periodontal tissue lesions, in the context of periodontal disease [28,34,53].

Therefore, the balance between antioxidant mechanisms and ROS is of utter importance in periodontal pathogenesis. Increased ROS and inhibited antioxidant mechanisms and/or decreased antioxidant capacity might lead to problems of the periodontium. Several authors reported a positive correlation between periodontal tissue damage and high levels of ROS [54,55,56,57]. Hypoxia and inflammation induced higher expression of ROS in primary periodontal ligament fibroblasts [55]. Besides, the exposure of periodontal ligament cells to hydrogen peroxide decreased their viability by promoting apoptosis [58].

Furthermore, animals infected with periodontal pathogens presented a five-fold increase in the OS index compared with controls [54]. Clinical studies also confirmed the link between ROS and progression of periodontitis [57]. A meta-analysis of 31 articles concluded that higher amounts of MDA and nitric oxide (NO) could be found in the peripheral blood of periodontal subjects [56]. Interestingly, the authors of the meta-analysis stated that SOD levels between normal and affected adults did not differ very much [56]. However, recent clinical studies encountered significantly lower levels of SOD in the serum [4] and gingival fluid [59] of periodontally diseased subjects. Of main importance are the results presenting higher amounts of total antioxidant capacity (TAC) [56,60] and CAT [61] in the serum of healthy patients. CAT is an enzyme that protects cells from hydrogen peroxide [61] and its decrease might be linked to failure of regulatory antioxidant mechanisms. Of real interest is also the fact that TAC [61] and SOD [4] levels rose significantly after periodontal treatment in the serum of periodontitis patients.

Researchers have also started to consider a possible relationship between periodontitis and systemic diseases. Diabetes mellitus [60,62,63], metabolic syndrome [64] and periodontal disease are all of related by a common factor known as OS. There was a major alteration of the local antioxidant defence mechanism in the gum and/or bone tissues of type 2 diabetes mellitus patients, which presented lower glutathione levels [62]. Interestingly, another clinical study concluded that adults affected by both periodontitis and diabetes mellitus exhibited higher serum values of SOD [60]. The authors considered that this might have been an adaptive mechanism against ROS that were developing in the tissues [60].

Further experiments demonstrated there is an increased amount of ROS not only in serum, but also in the oral fluids of periodontally diseased subjects [59,65]. Gingival crevicular fluid (GCF) of periodontitis sites exhibits a significantly greater total amount of GPx, lactoferrin, myeloperoxidase and interleukin-1beta (IL-1β) than healthy sites [66]. Moreover, MDA showed to be significantly up regulated in the GCF of adults with chronic and generalized aggressive periodontitis [59]. However, most importantly, saliva of periodontal patients often includes higher expression of oxidative and cellular energetic stress markers, increased purine degradation, GSH metabolism [65] and lower levels of uric acid. The low salivary levels of uric acid in periodontitis patients could be due to elevated rates of oral ROS in the context of chronic inflammatory reactions. Therefore, salivary uric acid might play an essential protective role against ROS and could be regarded as a local ROS marker in the context of chronic periodontitis [28,34,67].

New therapies are constantly being developed and could have future uses against OS present in chronic periodontitis: protein transduction treatments [68], bone targeted antiresorptives(bis-enoxacin and alendronate) [54] or antioxidants [57,58].

Ascorbic acid, an antioxidant, plays an important role in the maintenance of periodontal health in the elderly [69]. Its use led to really promising results *in vitro*, being able to partially antagonize the negative effects of hydrogen peroxide [58]. Furthermore, the administration of the antioxidant taurine in adults with chronic periodontitis resulted in a significant reduction of ROS present in plasma and gingival tissue, together with an improvement of periodontal status [57]. Other specific strategies could include lactic acid bacteria with antioxidative activity [70] or lipophilic antioxidants [71].

## 5. H_2_S—Involvement in Periodontal Diseases

Considering periodontitis a polymicrobial anaerobe infection, researchers focus on the following main mechanisms for explaining its pathogenesis: the production of certain waste products in the proteolytic metabolism, an intense host–inflammatory response and increased OS [2,3,29].

H_2_S is a bacterial waste product eliminated in the subgingival pocket [2,3] which, due to pro-inflammatory properties, might play an important role in the bacteria-induced inflammatory response in the periodontal diseases [72,73,74,75]. Other studies consider that oral malodorous compounds including H_2_S are causative agents of periodontitis because the toxicities are similar to that of cyanate [76]. However, H_2_S has also shown antioxidant properties, for example, in elevating endogenous antioxidase, such as SOD [8,77,78].

So, H_2_S can be regarded as a double-faced molecule: on one side, it can promote an antioxidant effect and becomes cytoprotective; while on the other side, it stimulates OS and is cytotoxic.

Reports show that H_2_S is directly linked to the initiation and development of periodontal diseases: the compound inhibits the proliferation process of oral keratinocyte cells [79], decreases protein synthesis in oral fibroblasts, and inhibits collagen synthesis or basal membrane synthesis [80].

There seems to be a direct relationship between the type of biological effect induced by H_2_S and the H_2_S levels. Low physiological concentrations of H_2_S have been shown to induce dental pulp cells differentiation towards hepatic or pancreatic cells [81] or to switch mouse osteoclasts from a passive state to an active one that induces bone resorption [81]. Moreover, physiological levels of endogenous H_2_S maintain the proliferation and differentiation of PDLSCs [14]. Blocking the endogenous H_2_S in PDLSCs led to significant reduction in their proliferation rate, as well as decreased osteogenic and adipogenic differentiation [14]. However, new H_2_S-releasing drugs had enhanced anti-inflammatory effects and reduced side effects in tissues [20]. Recently, a H_2_S -releasing derivative of naproxen, ATB-346, led to significant inhibition of alveolar bone loss and inflammation in periodontal rats [82].

High physiological concentrations of H_2_S are demonstrated to induce programmed cell death through different molecular pathways in a number of cell types. These concentrations induced apoptosis through inhibition of SOD in human gingival fibroblasts. This enzyme is paramount in eliminating ROS and leads to damage of DNA structure. The same process was observed in normal keratinocytes [83] and keratinocytes stem cells. DNA damage is shown to activate several molecular pathways such as p53 pathway that can decide cellular fate through activation of DNA repair molecules, cell cycle arrest or apoptosis.

Volatile sulfur compounds and especially H_2_S were shown to induce the apoptotic process in several cell types belonging to oral structures. Generally, apoptosis follows well established pathways such as: intrinsic mitochondrial pathway where the inner mitochondrial membrane is depolarized followed by cytochrome c release into cytosol, assembly of the apoptosome that leads to activation of initiator caspase 9 ultimately followed by executioner caspase 3 activation [84].

This pathway was activated in most studied cell types: oral fibroblasts, oral keratinocytes, oral keratinocyte stem cells, and general keratinocyte stem cells. The extrinsic pathway, or theligand-activated pathway was shown to be responsible for apoptosis induction only in cells isolated from the alveolar bone, osteoblast cells. At the same time, after H_2_S-exposure, human oral keratinocyte stem cells expressed key p53-related molecules associated with cell death, DNA repair and cell cycle control.

Another clinical effect of H_2_S is its association in the development of physiological and pathological halitosis [15,49]. Halitosis is the general term used to describe offensive smells detected in human breath and is a characteristic symptom of periodontal disease [15].

Several studies reported bacterial H_2_S producing species like *Fusobacteriumspp., Parvimonasmicra, Tannerella forsythia* or *Filifactoralocis* [2]. For example, *Fusobacteriumspp.* acts on substrates such as cysteine [85], homocysteine [86] or GSH [87]. Likewise, a recent study examined the GSH metabolism in *Treponemadenticola* [88].

Three steps has been proposed for the bacterial H_2_S production pathway. Glutamate or glutamine and the dipeptide cysteinylglycine (Cys-Gly), are obtained from GSH. Cys-Gly degradation results Gly and L-Cys. Pyruvate, ammonia, and H_2_S are the final products of L-Cys degradation. In GSH metabolism are involved three enzymes γ-glutamyltransferase (GGT), cysteinylglycinase, and L-cysteine desulfhydrase (cystalysin) [89,90], the last one cystalysin catalyzes the production of H_2_S, in the presence of L-cysteine [90,91]. 

The H_2_S -producing capacity is commonly tested with gas chromatography [89], colorimetric bismuth sulfide precipitation method [92], by using sensors [90] or by blackening of lead acetate paper [91].

In present more research is needed to be done under various conditions both *in vitro* and *in vivo* to detect the rate and amount of H_2_S produced by various species and strains. 

In addition to H_2_S, *Porphyromonas gingivalis*, produces several other virulence factors such as proteases (gingipains) [93], lipopolysaccharide (LPS) [94], and hemagglutinins [95]. A higher level of H_2_S (650–1.150 μmol/L), produced by *Porphyromonas gingivalis* affect IL-8 production in Phorbolmyristateacetat (PMA)-stimulated epithelial cells. 

A lower concentration of H_2_S (less than 400 μmol/L), did not effect IL-8 production in PMA-stimulated epithelial cells [75]. H_2_S present in blood at concentrations in the range of 30–100 μM [96], epithelial cells can accept H_2_S in concentrations lower than 400 μmol/L. Another study observed that increased levels of H_2_S (800 or 1.600 μmol/L) did not stimulate IL-8 production in epithelial cells in the absence of PMA [96].

This means that for H_2_S-mediated production of IL-8, the presence of a predisposed inflammatory condition is very important [75]. This could explain the dual behavior of H_2_S.Additionally, a recent study found that H_2_S synergistically up regulates *Porphyromonas gingivalis* LPS-induced expression of IL-6 and IL-8 in gingival fibroblasts and PDLCs, which could further promote the development of periodontitis [97]. Lower concentration of H_2_S inhibited LPS which induced synthesis of prostaglandin E2 (PGE_2_), NO, IL-1β and IL-6 in LPS-treated murine macrophages [98,99]. However, higher concentrations of NaHS, a H_2_S donor, promoted the synthesis of pro-inflammatory mediators [98,99]. An important function of IL-8 is chemo-attraction of neutrophils, which migrate to epithelial cells, the site where IL-8 is released and promote bacteria phagocytosis [100]. Neutrophils promote ROS production in order to kill bacteria cells but these ROS also seriously augment inflammation [101].

IL-8 production by epithelial cells is mediated by H_2_S, causes an enhancement of local inflammation by recruiting excess numbers of neutrophils. Several studies made *in vivo* on pancreas, liver and lung, observed that both endogenous H_2_S and exogenously supplied H_2_S increased neutrophil migration to the inflammation sites [102,103].

These data support the premise that the periodontal bacteria released H_2_S could induce the chemotaxis of neutrophils to the periodontal pocket, becoming this way, a real promoter of local OS, indirectly.

Exogenous H_2_S toxic effects in periodontal tissue has been showed [75], but the role of endogenous H_2_S in periodontal tissue physiologic function remains less understood. A recent study involving 43 subjects with moderate or severe periodontal breakdown could not correlate H_2_Sproduction to periodontal disease severity or to a specific bacterial composition [92]. It was suggested that H_2_Smay be a valuable clinical marker for degradation of proteins in the sub gingival pocket [92].

## 6. Conclusions

Altogether, data presented in recent studies suggests that the relationship between H_2_S, OS and periodontal diseases is controversial, but should not be underestimated. Further research is needed in order to elucidate the exact mechanisms and conditions which cause the H_2_S molecule to exhibit antioxidant or cytotoxic proprieties in the oral cavity.

To date, there is no general consensus regarding H_2_S biochemistry and its functions in cell biology (*i.e.*, its pro- or anti-inflammatory effects). In this respect, the field can be further expanded together with the development of tools that could correctly identify and quantify H_2_S synthesis and catabolism in organs and tissues [13]. Another important issue would be the elucidation of endo- or exogenous signals that initiate H_2_S production together with a better understanding of the chemical pathways responsible for its removal. Overall, a more clear understanding of the biochemistry of H_2_S in relation to its biological roles is greatly needed.
